# Bearing remaining useful life prediction based on optimized VMD and BiLSTM-CBAM*

**DOI:** 10.1371/journal.pone.0326399

**Published:** 2025-07-18

**Authors:** Wei Liu, Sen Liu

**Affiliations:** 1 School of Mechanical and Electrical Engineering, Zhoukou Normal University, Zhoukou, China; 2 School of Information and Control Engineering, Jilin Institute of Chemical Technology, Jilin, China; The University of British Columbia, AUSTRALIA

## Abstract

To address the issue of low accuracy in existing remaining useful life (RUL) prediction algorithms for rolling bearings, this paper proposes a novel RUL prediction method based on the Beluga Whale Optimization (BWO) algorithm, Variational Mode Decomposition (VMD), an improved Convolutional Block Attention Module (CBAM*), and a Bidirectional Long Short-Term Memory (BiLSTM) network. First, BWO is utilized to optimize the parameters of VMD, which is then applied to decompose and reconstruct the original vibration signals. Subsequently, time-domain and frequency-domain features are extracted from the reconstructed data to construct a degradation feature set. Finally, the degradation feature set is input into a BiLSTM network integrated with the CBAM* for RUL prediction of bearings. Comparative and ablation experiments are conducted on the IEEE PHM 2012 data set to evaluate the proposed method. The experimental results demonstrate that the method achieves lower Mean Absolute Error (MAE) and Root Mean Square Error (RMSE) in bearing RUL prediction compared to the other method models and ablation methods, highlighting the superiority and effectiveness of the proposed approach. This study not only ensures the safe operation of rotating machinery but also provides a valuable reference for RUL prediction of other types of equipment.

## 1. Introduction

Prognostics and Health Management (PHM) is an essential technology for the safe and reliable operation of production systems and is widely used in rotating machinery equipment [1]. Rolling bearings, as one of the key components of rotating machinery, operate in complex environments and are prone to degradation and failure. Their health status directly affects the safety of mechanical equipment. Therefore, the prediction of remaining useful life (RUL) plays a crucial role in performance degradation, faults, and sudden failures. This ensures the safe operation of machinery and facilitates predictive maintenance decisions [2].

RUL prediction techniques can be mainly categorized into three types: methods based on physics-of-failure models [3,4], methods based on probabilistic statistical models [5], and methods based on artificial intelligence [6]. Due to the complex and variable failure mechanisms of rotating machinery under diverse operating conditions, it is challenging to use physics-of-failure models for prediction. Methods based on probabilistic statistical models are often limited by the constraints of feature extraction and the inadequate expressive power of the models. Compared to methods based on physics-of-failure models and probabilistic statistical models, artificial intelligence-based methods primarily establish a spatial mapping relationship between condition monitoring data and RUL labels, without relying on physics-of-failure models and expert knowledge [7,8]. This provides valuable references for the RUL prediction of rolling bearings.

The first methods are based on physics-of-failure models. Sheng Y et al. [9] proposed a bearing RUL prediction method based on generalized high-order moments and an improved Paris-Erdogan model. This method uses the optimal health indicator as input to predict the remaining useful life after the initial failure of the bearing. Li G et al. [10] proposed a variational Bayesian and implicit Kalman filtering method to improve the prediction accuracy of rolling bearing RUL. Methods based on physics-of-failure models require the use of mathematical or physical models to analyze failure mechanisms [11,12], with the accuracy of the models depending on the understanding of these mechanisms. However, it is impossible to cover all influencing factors during the modeling process; thus, the final result is usually an approximation. In practical applications, obtaining mathematical or physical models for the operating process of many devices is neither economical nor feasible.

The second methods are based on probabilistic statistics. Wen L et al. [13] proposed a novel hybrid data and model approach—GRU-AE-Wiener. The bidirectional GRU model is structurally integrated with the Wiener process to form an autoencoder-like architecture, and the effectiveness of the method is validated on two datasets. Li W et al. [14] proposed an Integrated Dual-Scale Similarity-Based Prediction (IDS-SBP) method, which can fully extract degradation information from samples across two different time scales. Meng et al. [15] used fractal spectrum parameters of the first two intrinsic mode functions from differential empirical mode decomposition as performance degradation indicators and predicted these indicators using a combination of grey models and Markov processes. Hu et al. [16] predicted the RUL of wind turbine rolling bearings by using a Wiener process. This type of algorithm establishes a prediction model through mathematical theories such as stochastic processes and statistical inference, providing point estimates or prediction intervals for RUL. Probabilistic statistical methods have several drawbacks in bearing RUL prediction, including strong assumptions, lack of ability to model complex nonlinear relationships, reliance on large amounts of historical data, sensitivity to outliers, difficulty in handling multivariate problems, and insufficient reasoning capabilities, which limit their effectiveness in complex environments.

However, with the development of artificial intelligence technology, machine learning has been widely applied to the RUL prediction of rolling bearings [17–23]. GUO J et al. [24] employed Complete Ensemble Empirical Mode Decomposition with Adaptive Noise (CEEMDAN) and Kernel Principal Component Analysis (KPCA) to construct a nonlinear Health Indicator (HI). They then used a Dual-Channel Transformer network integrated with the Convolutional Block Attention Module (CBAM) to construct HIs for the remaining bearings. Finally, a nonlinear Wiener process with random effects was applied for degradation modeling and probabilistic Remaining Useful Life (RUL) prediction. Cheng et al. [25] proposed a dynamic domain adaptation method for predicting RUL under various conditions. This method uses fuzzy set theory to calculate conditional distribution differences and introduces dynamic adaptive factors to dynamically adjust distribution weights for RUL prediction. Liu et al. [26] proposed a bearing RUL prediction method based on the Long Short-Term Memory Network (LSTM), but unidirectional LSTM cannot utilize future information for prediction, which may lead to lower accuracy due to information limitations. Zheng Q et al. [27] divided the full life cycle of rolling bearings into a healthy stage and a degradation stage, and designed a domain invariant and domain-specific feature representation network (DIDSR) to predict the remaining useful life (RUL) of rolling bearings. Liu et al. [28] proposed a Vector-Weighted Fusion (V-DWF) algorithm for dynamically evaluating the sensitivity of each feature over time and assigning sensitivity weights to corresponding features to estimate RUL. Zhu et al. [29] first preprocessed the original signal using Complete Ensemble Empirical Mode Decomposition with Adaptive Noise (CEEMDAN) and then proposed a novel RLSTM network for RUL prediction. Wei et al. [30] combined Graph Convolutional Networks with self-attention mechanisms for predicting the RUL of rolling bearings. These methods have all achieved good prediction results, which are of significant practical importance for management and decision-making activities within the PHM framework, such as spare parts ordering and maintenance scheduling. AI-based methods in bearing RUL prediction have advantages such as automatic feature extraction, strong adaptability, the ability to handle complex data, less reliance on prior knowledge, high prediction accuracy, and strong real-time performance and robustness, providing accurate and reliable predictions in complex and dynamic environments.

In response to the current issue of low accuracy in Remaining Useful Life (RUL) prediction methods for rolling bearings, this paper proposes a novel RUL prediction approach based on Bidirectional Long Short-Term Memory (BiLSTM) networks, Convolutional Block Attention Module (CBAM), Beluga Whale Optimization (BWO) algorithm, and Variational Mode Decomposition (VMD). The main contributions of this paper are as follows:

(1) The parameters of variational mode decomposition (VMD) are optimized using the white whale optimization algorithm, and the vibration signal of the rolling bearing is decomposed and denoised.
(2) A comprehensive evaluation method in the time-frequency domain is proposed based on monotonicity and correlation. After evaluating the extracted time-frequency domain features, they are filtered to extract a feature set that strongly reflects the degradation trend of the rolling bearing.
(3) The convolutional attention mechanism is improved by replacing the spatial attention mechanism in the traditional convolutional attention mechanism with a temporal attention mechanism, making it more suitable for time series prediction.

**Fig 1:**
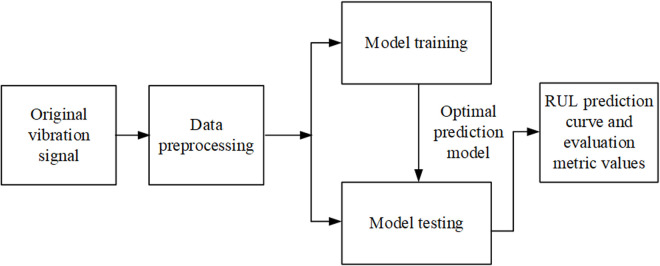
Research Framework. The proposed framework for rolling bearing RUL prediction.

The framework of the research conducted in this study is shown in Fig 1.

The original signal undergoes signal preprocessing and is divided into model training and model testing. During the model training stage, the optimal RUL prediction model is obtained, which is then used for the RUL prediction of rolling bearings during the testing stage. After the testing stage, the bearing RUL prediction curve and evaluation metric values are obtained.

## 2. BWO optimizes VMD parameters

### 2.1 Variational mode decomposition

Variational Mode Decomposition (VMD) was proposed by Dragomiretskiy et al. [31] in2014. It is a method that decomposes an input signal into a finite number of sub-modes. The steps of VMD are as follows:

**Step 1:** Assuming that VMD decomposes the original signal 𝑓 into 𝑘 intrinsic mode functions (IMFs), we have:


$$\begin{array}{c} \min_{u_{k},\left\{\omega_{k}\right\}}\;\left\{\sum {\mathrm{\backslash nolimits}}_{k}\;∥\partial_{t}\left[\left(\delta (t)+\frac{j}{\pi t}\right)*u_{k}(t)\right]e^{-j\omega_{k}t}{∥}_{2}^{2}\right\} \end{array}$$ (1)

Where $\begin{array}{c} A_{k}(\begin{array}{c} t \end{array}) \end{array}$ is the instantaneous amplitude of the mode, $\phi_{k}(\begin{array}{c} t \end{array})$ is the non-decreasing phase function of the mode, and $u_{k}$ is the *k*-*th* mode function.

**Step 2:** Perform the Hilbert transform on each IMF and calculate its single-sided spectrum, then modulate its spectrum to the corresponding baseband. By calculating the squared norm *L*^*2*^ of the demodulated gradient, the bandwidth of each IMF is estimated. The mathematical model is as follows:


$$\begin{array}{c} \min_{u_{k},\left\{\omega_{k}\right\}}\;\left\{\sum {\mathrm{\backslash nolimits}}_{k}\;∥\partial_{t}\left[\left(\delta (t)+\frac{j}{\pi t}\right)*u_{k}(t)\right]e^{-j\omega_{k}t}{∥}_{2}^{2}\right\} \end{array}$$ (2)

Where $u_{k}$ is the *k*-th mode function, *denotes the convolution operation, $\omega_{k}$ is the center frequency of the *k*-th IMF component, δ(t) is the Dirac function.

**Step 3:** By introducing a quadratic penalty factor 𝛼 and a Lagrange multiplier λ, the constrained variational problem is converted into an unconstrained variational problem for solving:


$${\hat{u}}_{k}^{n+1}(\omega )=\frac{\hat{f}(\omega )-\sum_{i<k}\;{\hat{u}}_{k}^{n+1}(\omega )-\sum_{i>k}\;{\hat{u}}_{k}^{n}(\omega )+\frac{{\hat{\lambda }}^{n}(\omega )}{2}}{1+2\alpha {\left(\omega -\omega_{n}^{k}\right)}^{2}}$$ (3)

**Step 4:** Use the alternating direction method of multipliers (ADMM) for optimization. During the process of finding the optimal solution, continuously update the mode functions $u_{k}^{n+1}$, center frequencies $\omega_{k}^{n+1}$, and Lagrange multipliers $\lambda_{k}^{n+1}$. The specific steps are as follows:


$${\hat{u}}_{k}^{n+1}(\omega )=\frac{\hat{f}(\omega )-\sum_{i<k}\;{\hat{u}}_{k}^{n+1}(\omega )-\sum_{i>k}\;{\hat{u}}_{k}^{n}(\omega )+\frac{{\hat{\lambda }}^{n}(\omega )}{2}}{1+2\alpha {\left(\omega -\omega_{n}^{k}\right)}^{2}}$$ (4)

$$\omega_{k}^{n+1}=\frac{\int_{0}^{\infty }\;\omega |{\hat{u}}_{k}^{n+1}(\omega ){|}^{2}dw}{\int_{0}^{\infty }\;|{\hat{u}}_{k}^{n+1}(\omega ){|}^{2}dw}$$ (5)

$${\hat{\lambda }}_{k}^{n+1}(\omega )={\hat{\lambda }}_{k}^{n}(\omega )+\tau \left(\hat{f}(\omega )-\sum_{k}\;{\hat{u}}_{k}^{n+1}(\omega )\right)$$ (6)

**Step 5:** Repeat the above steps until the updates in the mode functions and center frequencies become very small, indicating convergence.

### 2.2 Beluga whale optimization algorithm

The Beluga Whale Optimization (BWO) algorithm is a bio-inspired optimization algorithm based on the behavior of beluga whales. This algorithm was proposed by Zhong et al. [32] in 2022. BWO simulates the social, survival, and foraging behaviors of beluga whales to help optimize various data parameters.

The BWO optimization algorithm has significant advantages over Particle Swarm Optimization (PSO), Genetic Algorithm (GA), and Ensemble Empirical Mode Decomposition (EEMD). Firstly, BWO offers faster convergence speed and higher search efficiency, enabling it to find better solutions with fewer iterations and avoid getting trapped in local optima. Secondly, BWO achieves higher optimization accuracy and better stability. Compared with PSO and GA, which are often sensitive to parameter settings, BWO can more accurately obtain the optimal parameter combination for VMD. Finally, the BWO algorithm features a simple structure and few parameters, making it easier to implement and debug, thus well-suited for practical engineering applications.

The BWO algorithm optimizes based on the population, with each beluga whale representing a candidate solution in the population. During the evolutionary process of the algorithm, the positions of the beluga whales are continuously updated. The initial positions of the beluga whale population are modeled as follows:


$$X=[\begin{array}{cc} x_{1,1}x_{1,2} & ...x_{1,d}\\ x_{2,1}x_{2,2} & ...x_{2,d}\\ \vdots \vdots & \vdots \vdots \\ x_{n,1}x_{n,2} & ...x_{n,d} \end{array}]$$ (7)

where *n* is the size of the beluga whale population and *d* is the dimensionality of the variables.

The BWO algorithm primarily consists of the exploration phase, exploitation phase, and whale fall phase.

1) **Exploration phase**

The exploration phase is modeled based on the swimming behavior of beluga whales. The position update formula for the beluga whales is as follows:


$$X_{i}^{T}$$ (8)

Where *T* represents the total number of iterations for the current population, $X_{i,j}^{t+1}$ denotes the position of the *i*-th beluga whale in the *j*-th dimension, $p_{j}$ is a randomly chosen index from *d* dimensions, *r* denotes a randomly selected beluga whale, $r_{1}$ and $r_{2}$ are random numbers between 0 and 1, $X_{i,p_{j}}^{t}$ and $X_{r,p_{1}}^{t}$ represent the positions of the *i*-th and *r*-th beluga whales, respectively, at the *T*-th iteration, $\sin(2\pi r_{2})$ and $cos(2\pi r_{2})$ represent the orientation of the mirror image whale’s fins relative to the water surface.

2) **Exploitation phase**

The exploitation phase is modeled based on the foraging behavior of beluga whales. By introducing the *Levy* flight strategy, the algorithm’s local search performance is improved. The position update formula for the beluga whales is as follows.


$$X_{i}^{T}$$ (9)

Where $X_{i}^{T}$ and $X_{r}^{T}$ represent the current positions of the *i*-th beluga whale and the randomly chosen beluga whale, respectively, $X_{best}^{T}$ denotes the optimal position of the beluga whale; $X_{i}^{T+1}$ is the updated position of the *i*-th beluga whale, $r_{3}$ and $r_{4}$ are random numbers between 0 and 1, $C_{1}$ is the random jump intensity for Levy flight, where $C_{1}=2r_{4}(1-T/T_{\max})$, u and ν are random numbers following a normal distribution, and β is a constant, where β=1.5.

3) **Whale fall phase**

When beluga whales face external threats, they change their current positions. To ensure that the total population remains constant, the positions of the beluga whales are updated based on their current positions and the fall step size. The position update formula is as follows:


$$r_{5}$$ (10)

Where $r_{5}$, $r_{6}$, and $r_{7}$ are random numbers between 0 and 1, $X_{step}$ denotes the fall step size of the beluga whale, $u_{b}$ and $l_{b}$ represent the upper and lower bounds of the search, $C_{2}$ is the step size factor, which is related to the fall probability and the population size, $W_{f}$ denotes the probability of a beluga whale falling.

### 2.3 BWO Optimizes VMD

**Fig 2:**
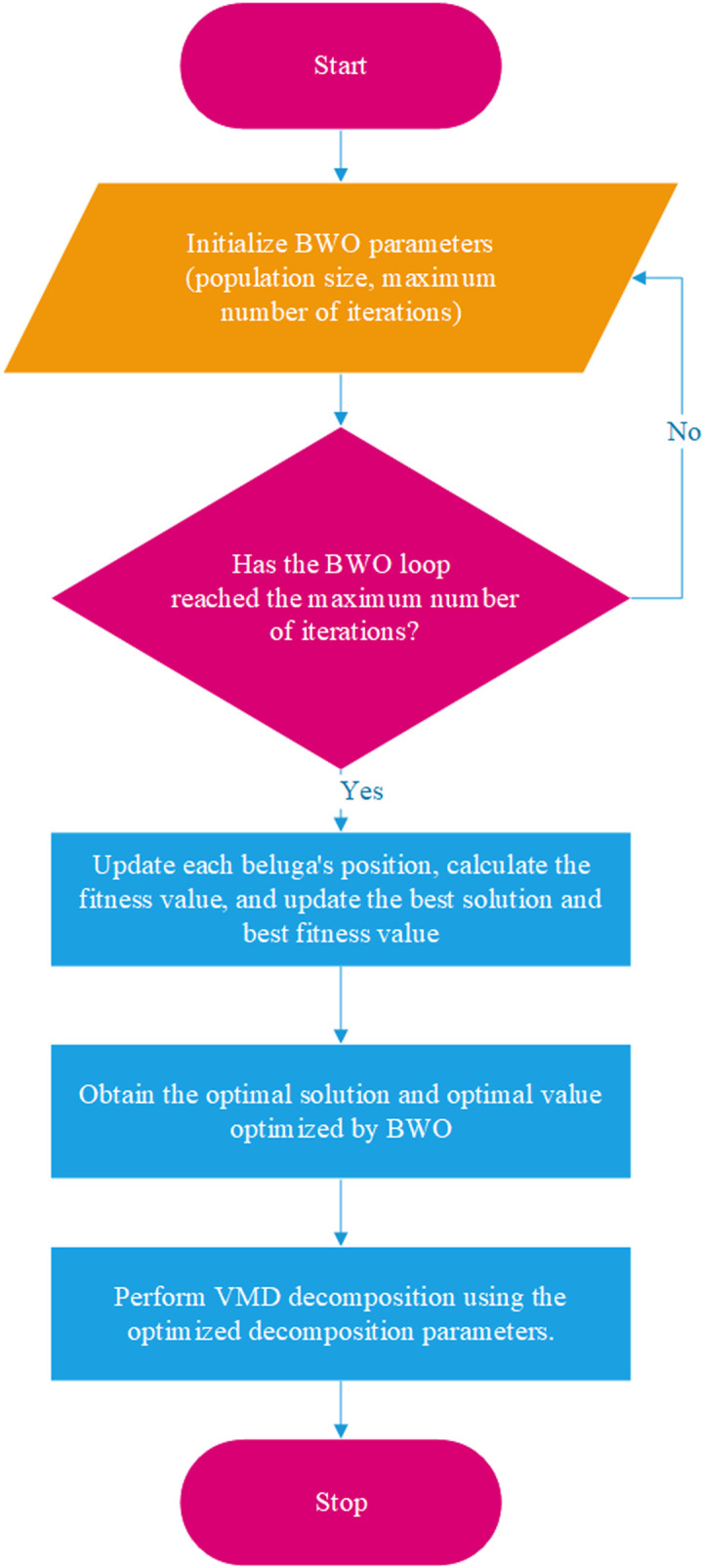
Flowchart of BWO optimized VMD. The process of optimizing the decomposition parameters of VMD using the BWO optimization algorithm.

The flowchart of the Beluga Whale Optimization (BWO) algorithm for optimizing VMD parameters proposed in this study is shown in Fig 2.

First, the population size and the maximum number of iterations are initialized. Then, the whale population is initialized, the initial fitness values are calculated, and the best solution is recorded. Next, the BWO optimization loop is performed: if the maximum number of iterations is reached, the position of each whale is updated, fitness values are calculated, and the best solution and best fitness value are updated. Finally, the optimal solution and value obtained by BWO optimization are retrieved. The range for k is set to [2,10], the range for α is set to [100, 5000], and the number of iterations is set to 100.

## 3. Rolling bearing RUL prediction method

### 3.1 Bidirectional long short-term memory network

**Fig 3:**
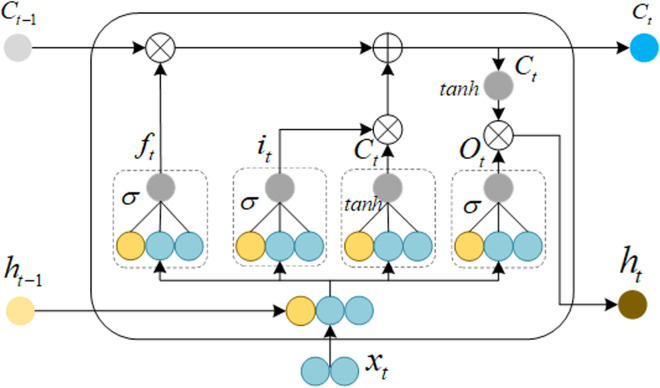
LSTM network structure. Long Short-Term Memory network architecture.

The output from each hidden unit at the previous time step of an RNN (Recurrent Neural Network) is used as the input for the prediction unit at the next time step. However, due to the gradient vanishing problem in RNNs, they are not suitable for predicting long data sequences. LSTM (Long Short-Term Memory) is a neural network with memory capabilities that adds mechanisms for storing long-term dependencies and selectively forgetting information on the basis of RNNs. In comparison, LSTM networks control the flow of information through gating units, effectively mitigating the gradient vanishing problem. Therefore, they are more suitable for predicting long sequence data [17,33]. The structure of an LSTM is shown in Fig 3.

The principles of the LSTM model are as follows:

(1) The forget gate is responsible for selecting the information to be discarded:

$$f_{t}=\sigma \left(W_{f}[h_{t-l},x_{t}]+b_{f}\right)$$ (11)

Where $f_{t}$ is the weight value for retaining information, σ is the sigmoid activation function, $W_{f}$ represents the weight value of the corresponding network, $h_{t-l}$ is the hidden layer information from the previous time step, $x_{t}$ is the input at the current time step, and $b_{f}$ is the bias of the corresponding network.

(2) The input gate is responsible for identifying the information to be saved:

$$C_{t}=f_{t}*C_{t-1}+\sigma \left(W_{i}[h_{t-1},x_{t}]+b_{i}\right)*tanh\left(W_{c}[h_{t-1},x_{t}]+b_{c}\right)$$ (12)

Where tanh is the activation function, $C_{t}$ is the current cell state information, $C_{t-1}$ is the previous cell state information, and * represents the dot product operation.

(3) The output gate is responsible for determining the information to be output:

$$h_{t}=\sigma (W_{o}[h_{t-1,x_{t}}]+b_{o})*tanh(c_{t})$$ (13)

Where $h_{t}$ represents the hidden information.

**Fig 4:**
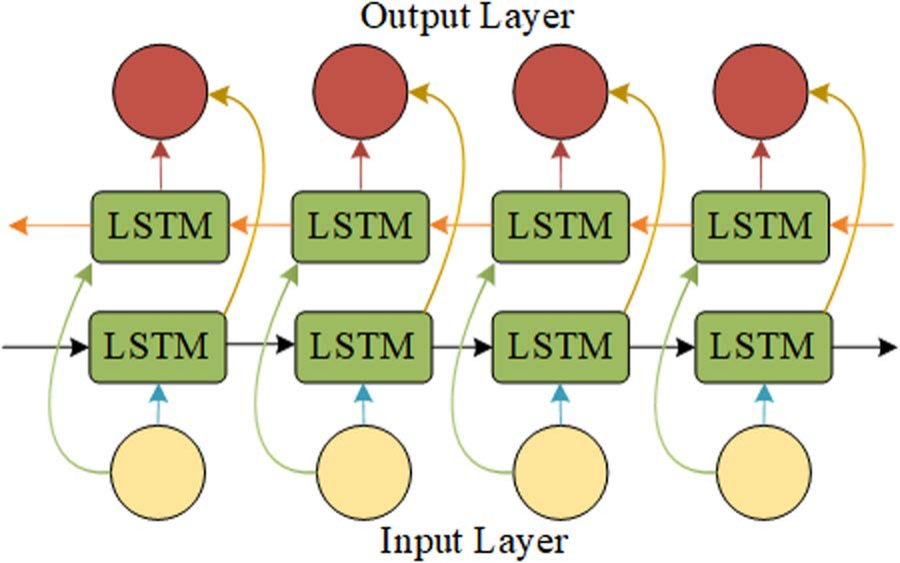
BiLSTM network structure. Bidirectional Long Short-Term Memory network architecture.

Although Long Short-Term Memory (LSTM) networks can address the issue of long-term dependencies, unidirectional LSTM networks can only propagate information in one direction and cannot utilize future information, potentially leading to limited information. Bidirectional Long Short-Term Memory (BiLSTM) networks, on the other hand, can consider both historical and future data simultaneously, thereby resolving this issue. The structure of a Bidirectional Long Short-Term Memory network is shown in Fig 4.

The forward LSTM and backward LSTM respectively capture the historical information and new information from the future data in the input sequence, thereby enhancing the model’s prediction performance. Therefore, we introduce the BiLSTM network as the backbone network for rolling bearing RUL prediction.

### 3.2 Convolutional block attention module

Compared to the standard self-attention mechanism, CBAM has the advantages of being lightweight and having strong generalization capability. Additionally, the channel attention mechanism can identify important feature dimensions, enhance useful information, and suppress irrelevant noise.

The traditional Convolutional Block Attention Module (CBAM) consists of a channel attention mechanism and a spatial attention mechanism [34]. The spatial attention mechanism is more suitable for processing two-dimensional images, whereas the full life-cycle signals of rolling bearings are one-dimensional data evolving over time. Compared to the spatial attention mechanism, the temporal attention mechanism can more effectively capture the dynamic temporal variations and trends within rolling bearing data, thereby improving the accuracy and robustness of the predictive model for the remaining useful life of rolling bearings.

Based on the above analysis, this paper replaces the spatial attention mechanism in the traditional CBAM with a temporal attention mechanism. The specific computation process of the temporal attention mechanism is as follows:


$$Q=XW^{q}$$ (14)

$$K=XW^{k}$$ (15)

$$V=XW^{\nu }$$ (16)

$$E=QK^{T}/\sqrt{d_{k}}$$ (17)

A=softmax(E) (18)

O=AV (19)

Where Q is the query matrix, K is the key matrix, V is the value matrix, $W^{q}$、$W^{k}$ and $W^{\nu }$ are the weight matrices, X is the input vector, *d*_*k*_ is the dimensionality of Q, K and V, E represents the attention scores, A denotes the attention weights, and O is the final output.

**Fig 5:**
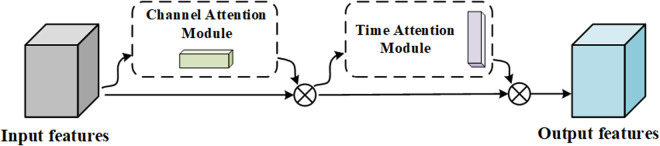
CBAM* network structure. The improved CBAM, named CBAM*.

The CBAM^*^ network structure is shown in Fig 5.

CBAM* replaces the spatial attention mechanism in the original CBAM structure with a temporal attention mechanism. The temporal attention mechanism can better capture long-term dependency features and has advantages in long-sequence prediction tasks. Therefore, CBAM* is better suited for processing rolling bearing vibration data prediction.

### 3.3 Feature extraction and degradation feature set construction

By extracting different feature parameters, a comprehensive representation of the rolling bearing’s operating condition degradation and obtaining the equipment’s health status can be achieved. However, the correlation and redundancy among feature parameters can lead to high computational complexity in the prediction model and affect the accuracy of the prediction results. Therefore, in addition to extracting various operational state feature parameters from complex vibration signals, features that are sensitive to operating condition changes and have strong health status representation capabilities should be further selected based on comprehensive evaluation methods. This approach aims to improve prediction efficiency and reduce the computational complexity of the network model [35].

The time-domain features extracted in this study are shown as *T*_*1*_ to *T*_*11*_ in Table 1, The frequency-domain features extracted after performing the Fast Fourier Transform are shown as *F*_*1*_ to *F*_*13*_ in Table 1.

**Table 1:** Time domain and frequency domain features. The extracted 24 time-domain and frequency-domain features.

Feature	Formula		Feature			Formula		Feature		Formula	Feature		Formula		Feature			Formula
*T*_*1*_		$\frac{\text{1}}{\text{N}}\sum_{\text{n=1}}^{\text{N}}\text{\textbackslash{},}{\text{x}}_{\text{n}}$		*T*_*2*_	$\frac{\text{1}}{\text{N-1}}\sum_{\text{n=1}}^{\text{N}}\text{\textbackslash{},}({\text{x}}_{\text{n}}\text{-}{\text{T}}_{\text{1}}\text{)}$		*T*_*3*_		${\left[\frac{\text{1}}{\text{N}}\sum_{\text{n=1}}^{\text{N}}\text{\textbackslash{},}\sqrt{\left|{\text{x}}_{\text{n}}\right|}\right]}^{\text{2}}$			*T*_*4*_		$\sqrt{\frac{\text{1}}{\text{N}}\sum_{\text{n=1}}^{\text{N}}\text{\textbackslash{},}{\text{x}}_{\text{n}}^{\text{2}}}$		*T*_*5*_	$\text{max}\left\{\left|{\text{x}}_{\text{n}}\right|\right\}$	
*T*_*6*_		$\frac{\frac{\text{1}}{\text{N}}\sum_{\text{n=1}}^{\text{N}}\text{\textbackslash{},}{\text{(}{\text{x}}_{\text{n}}\text{-}{\text{T}}_{\text{1}}\text{)}}^{\text{3}}}{{\text{T}}_{\text{4}}^{\text{3}}}$		*T*_*7*_	$\frac{\frac{\text{1}}{\text{N}}\sum_{\text{n=1}}^{\text{N}}{\text{(}{\text{x}}_{\text{n}}\text{-}{\text{T}}_{\text{1}}\text{)}}^{\text{4}}\text{\textbackslash{},}}{{\text{T}}_{\text{4}}^{\text{4}}}$		*T*_*8*_		$\frac{{\text{T}}_{\text{5}}}{{\text{T}}_{\text{4}}}$			*T*_*9*_		$\frac{{\text{T}}_{\text{5}}}{{\text{T}}_{\text{3}}}$		*T*_*10*_	$\frac{{\text{T}}_{\text{4}}}{\frac{\text{1}}{\text{N}}\sum_{\text{n=1}}^{\text{N}}\text{\textbackslash{},}|{\text{x}}_{\text{n}}\text{|}}$	
*T*_*11*_		$\frac{{\text{T}}_{\text{5}}}{\frac{\text{1}}{\text{N}}\sum_{\text{n=1}}^{\text{N}}\text{\textbackslash{},}|{\text{x}}_{\text{n}}\text{|}}$		*F*_*1*_	$\frac{\text{1}}{\text{K}}\sum_{\text{k=1}}^{\text{K}}\text{\textbackslash{},}{\text{S}}_{\text{k}}$		*F*_*2*_		$\frac{\sum_{\text{k=1}}^{\text{K}}\text{\textbackslash{},}({\text{S}}_{\text{k}}\text{-}{\text{F}}_{\text{1}}{\text{)}}^{\text{2}}}{\text{K-1}}$			*F*_*3*_		$\frac{\sum_{\text{k=1}}^{\text{K}}\text{\textbackslash{},}({\text{S}}_{\text{k}}\text{-}{\text{F}}_{\text{1}}{\text{)}}^{\text{3}}}{\text{K}(\sqrt{{\text{F}}_{\text{2}}}{\text{)}}^{\text{3}}}$		*F*_*4*_	$\frac{\sum_{\text{k=1}}^{\text{K}}\text{\textbackslash{},}({\text{S}}_{\text{k}}\text{-}{\text{F}}_{\text{1}}{\text{)}}^{\text{4}}}{\text{K}{\text{F}}_{\text{2}}^{\text{2}}}$	
*F*_*5*_	$\frac{\sum_{\text{k=1}}^{\text{K}}\text{\textbackslash{},}{\text{f}}_{\text{k}}{\text{S}}_{\text{k}}}{{\text{S}}_{\text{k}}\sum_{\text{k=1}}^{\text{K}}\text{\textbackslash{},}}$		*F*_*6*_			$\sqrt{\frac{\sum_{\text{k=1}}^{\text{K}}\text{\textbackslash{},}({\text{f}}_{\text{k}}\text{-}{\text{F}}_{\text{5}}{\text{)}}^{\text{2}}{\text{S}}_{\text{k}}}{\text{K}}}$		*F*_*7*_		$\sqrt{\frac{\sum_{\text{k=1}}^{\text{K}}\text{\textbackslash{},}{\text{f}}_{\text{k}}^{\text{2}}{\text{S}}_{\text{k}}}{\sum_{\text{k=1}}^{\text{K}}\text{\textbackslash{},}{\text{S}}_{\text{k}}}}$	*F*_*8*_		$\sqrt{\frac{\sum_{\text{k=1}}^{\text{K}}\text{\textbackslash{},}{\text{f}}_{\text{k}}^{\text{4}}{\text{S}}_{\text{k}}}{\sum_{\text{k=1}}^{\text{K}}\text{\textbackslash{},}{\text{f}}_{\text{k}}^{\text{2}}{\text{S}}_{\text{k}}}}$		*F*_*9*_			$\frac{\sum_{\text{k=1}}^{\text{K}}\text{\textbackslash{},}{\text{f}}_{\text{k}}^{\text{2}}{\text{S}}_{\text{k}}}{\sqrt{\sum_{\text{k=1}}^{\text{K}}{\text{S}}_{\text{k}}\sum_{\text{k=1}}^{\text{K}}\text{\textbackslash{},}{\text{f}}_{\text{k}}^{\text{4}}{\text{S}}_{\text{k}}}}$
*F*_*10*_	$\frac{{\text{F}}_{\text{6}}}{{\text{F}}_{\text{5}}}$		*F*_*11*_			$\frac{\sum_{\text{k=1}}^{\text{K}}\text{\textbackslash{},}({\text{f}}_{\text{k}}\text{-}{\text{F}}_{\text{5}}{\text{)}}^{\text{3}}{\text{S}}_{\text{k}}}{\text{K}{\text{F}}_{\text{6}}^{\text{3}}}$		*F*_*12*_		$\frac{\sum_{\text{k=1}}^{\text{K}}\text{\textbackslash{},}({\text{f}}_{\text{k}}\text{-}{\text{F}}_{\text{5}}{\text{)}}^{\text{4}}{\text{S}}_{\text{k}}}{\text{K}{\text{F}}_{\text{6}}^{\text{4}}}$	*F*_*13*_		$\frac{\sum_{\text{k=1}}^{\text{K}}\text{\textbackslash{},}\sqrt{\text{|}{\text{f}}_{\text{k}}\text{-}{\text{F}}_{\text{5}}\text{|}}{\text{S}}_{\text{k}}}{\text{K}\sqrt{{\text{F}}_{\text{6}}}}$					

In the Table 1, $x_{n}$ represents the time-domain signal sequence, where n=1,2,...,N, with *N* being the number of sampling points for each signal. $S_{k}$ represents the frequency-domain signal sequence, where K=1,2,...,K, with K being the number of spectral lines. $f_{k}$ denotes the frequency value of the *k*-th spectral line.

During the degradation process of bearings, time-frequency domain features can effectively reflect the degradation. However, different time-frequency domain features exhibit significant differences in their ability to reflect the degradation trend. To accurately extract features that clearly indicate the degradation trend, it is necessary to filter the extracted time-domain and frequency-domain features. A single metric cannot fully evaluate the applicability of the degradation features. Therefore, this paper uses a linear combination of monotonicity (Mon) and correlation (Cor) as a comprehensive evaluation method for degradation features, expressed as follows.

To determine the weight assignment for monotonicity (Mon) and correlation (Cor), we conducted RUL prediction experiments on bearing 2_4 by assigning different weight values to Mon and Cor. We then compared the mean absolute error (MAE) and root mean square error (RMSE) to identify the optimal weight combination. The RUL prediction errors corresponding to different weight values are shown in Table 2.

**Table 2:** RUL prediction errors corresponding to equal weight values. The effect of varying feature evaluation weights on the prediction error.

Monotonicity weight value	Correlation weight value	MAE	RMSE
0.3	0.7	0.064	0.076
0.4	0.6	0.052	0.061
**0.5**	**0.5**	**0.040**	**0.055**
0.6	0.4	0.062	0.077
0.7	0.3	0.085	0.096

As observed from Table 2. When the monotonicity weight and correlation weight are both set to 0.5, the MAE and RMSE reach their minimum values. Therefore, the comprehensive feature evaluation expression is defined as follows.


Ce=0.5Mon+0.5Cor (20)

**Fig 6:**
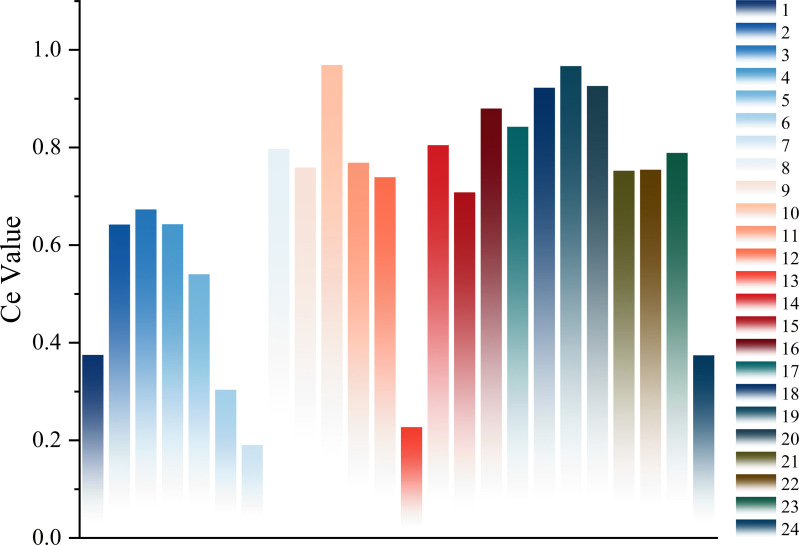
Comprehensive evaluation value of the 24 features of Bearing 2_4. The results obtained by evaluating the features of bearing 2_4 using the proposed comprehensive feature evaluation method.

**Fig 7:**
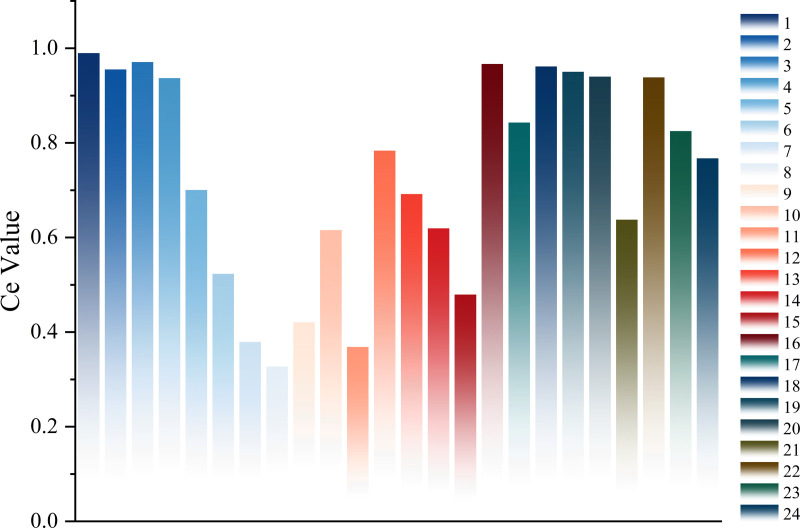
Comprehensive evaluation value of the 24 features of Bearing 2_7. The results obtained by evaluating the features of bearing 2_7 using the proposed comprehensive feature evaluation method.

Using bearings 2_4 and 2_7 as examples, we evaluate the 24 extracted time-domain and frequency-domain features using Eq. (20). The comprehensive evaluation value of Bearing 2_4 is shown in Fig 6, and that of Bearing 2_7 is shown in Fig 7.

As shown in Fig 6 and Fig 7. Different features receive varying scores after the comprehensive evaluation. Features with a comprehensive evaluation value below 0.5 have a weaker ability to reflect the degradation trend of rolling bearings [36]. Therefore, this study retains only the degradation features with scores above 0.5 to construct the degradation feature set for rolling bearings.

### 3.4 The RUL prediction method proposed in this paper

**Fig 8:**
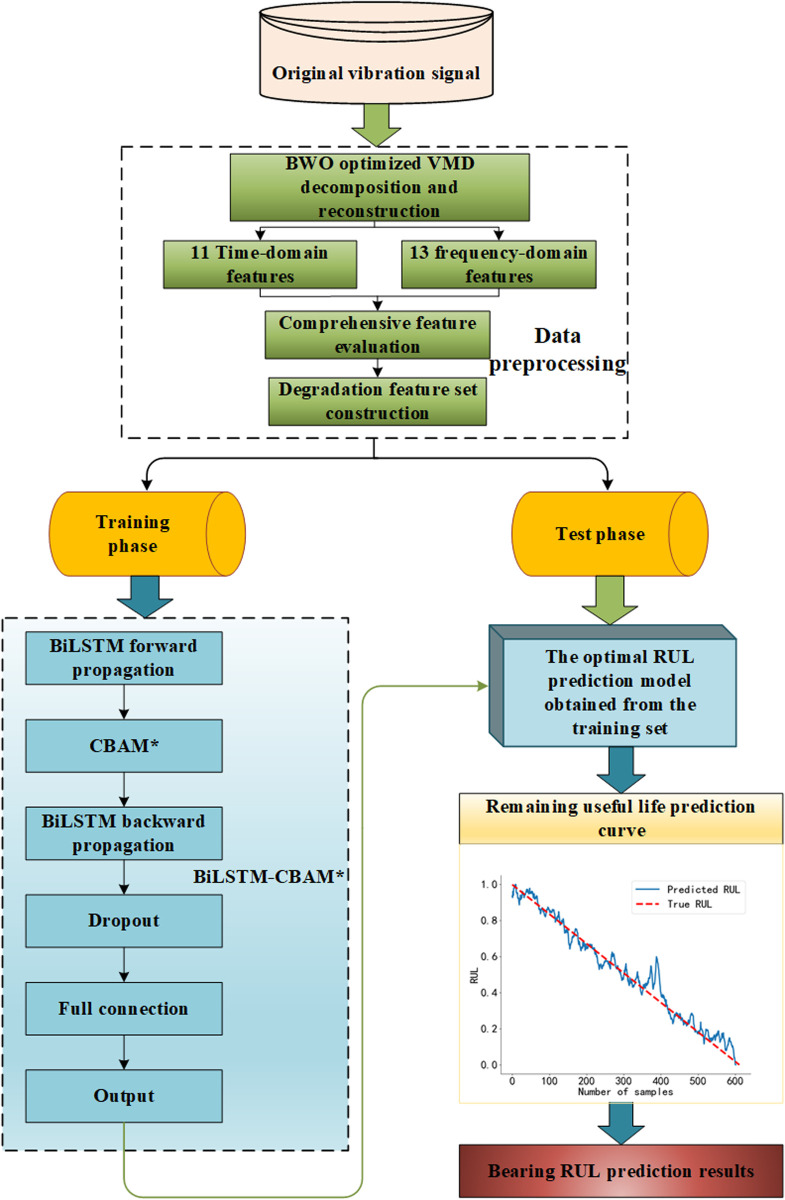
Prediction process diagram. The proposed method for predicting the remaining useful life of rolling bearings.

The RUL prediction process for rolling bearings based on optimized VMD and BiLSTM-CBAM^*^ mechanism proposed in this study is shown in Fig 8.

As illustrated in Fig 8. The methodology proposed in this study is divided into three main stages: data preprocessing, model training, and model evaluation.

Data Preprocessing Stage. Initially, the original vibration signals are decomposed, reconstructed, and denoised using a BWO-optimized Variational Mode Decomposition (VMD) algorithm. Subsequently, 24 time-domain and frequency-domain features are extracted from the reconstructed and denoised signals. These features, listed in Table 1, are then comprehensively evaluated using Equation (20). Features with a comprehensive evaluation score exceeding 0.5 are selected to form the degradation feature set for rolling bearings, thereby reducing the prediction error associated with Remaining Useful Life (RUL) estimation.

Model Training Stage. To enhance the feature representation capabilities in both the forward and backward directions, the CBAM* module is integrated between the forward-propagating and backward-propagating layers of the BiLSTM network. This integration facilitates more effective modeling of long-term dependencies and improves prediction accuracy. Furthermore, a dropout mechanism is employed to mitigate the risk of network overfitting. The resulting predictive model is denoted as BiLSTM-CBAM*.

Model Evaluation Stage. The degradation feature set obtained from the preprocessed test data is input into the optimal BiLSTM-CBAM* model trained on the training set to perform RUL prediction. Normalized data are used as the true labels for RUL. During the bearing degradation process, a fitting function is utilized to model the degradation trajectory of the predicted features. The real-time RUL prediction model is continuously updated by fitting newly acquired data points and capturing the evolving degradation trend of each point.

## 4. Experiments and results analysis

### 4.1 Dataset description

**Fig 9:**
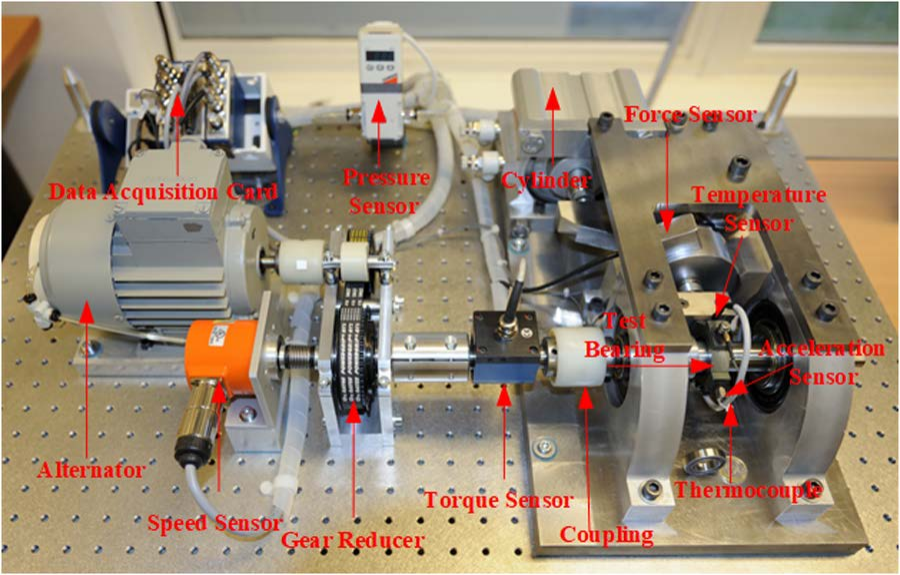
PRONOSTIA test platform. The experimental platform for data acquisition of the IEEE PHM 2012 dataset.

The bearing vibration data used in this study comes from the 2012 PHM Data Challenge [21,37], which was initiated by the Institute of Electrical and Electronics Engineers (IEEE). The structure of the PRONOSTIA test platform is shown in Fig 9.

**Fig 10:**
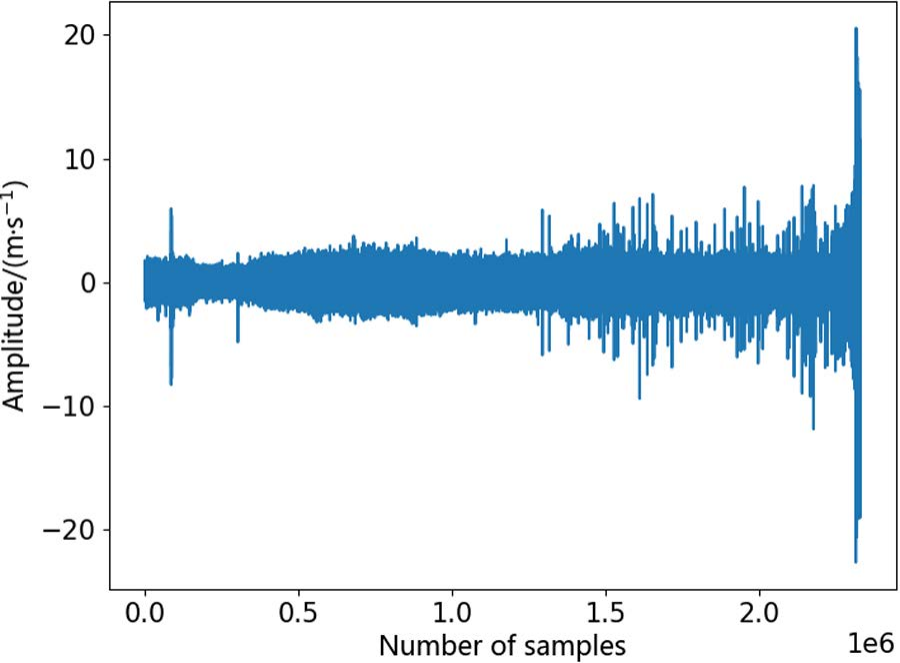
Full lifecycle diagram of bearings2_1. Vibration acceleration signal diagram of bearing 2_1 over its full lifecycle from the PHM2012 dataset.

As shown in Fig 9. The PRONOSTIA test platform primarily consists of four parts: the motor, the load section, the tested bearing, and various sensors. This experimental platform can accelerate bearing degradation under constant or variable conditions and collects temperature and vibration acceleration information in both horizontal and vertical directions. At the start of data collection, all bearings are healthy and defect-free. Data collection stops when the vibration amplitude reaches the fault threshold of the bearing. The full lifecycle vibration signal of bearing 2_1 is shown in Fig 10.

By observing the full lifecycle signal plot of bearing 2_1, the vibration signal transitions from steady vibrations during the healthy stage of the bearing to intense vibrations at complete failure.

The IEEE PHM 2012 dataset includes full lifecycle data for rolling bearings under three different operating conditions. Operating conditions 1 and 2 each contain full lifecycle data for 7 bearings, while operating condition 3 contains full lifecycle data for 3 bearings. The rotational speeds and loads for each operating condition are different, as detailed in Table 3.

**Table 3:** IEEE PHM 2012 dataset. The distribution of the IEEE PHM 2012 dataset.

Operating Condition Number	Speed (RPM)	Load (N)	Number of Bearing Samples
1	4000	1800	7
2	4200	1650	7
3	5000	1500	3

Since horizontal vibration signals contain more degradation information compared to vertical vibration signals [12], only horizontal vibration signals are used to validate the effectiveness of the proposed method. Compared to Condition 3, Conditions 1 and 2 have more sample data, making the prediction results more representative. Therefore, this study only uses data samples from Conditions 1 and 2.

### 4.2 Data processing and evaluation metrics

#### 4.2.1 Data processing and label setting.

In the full-life vibration signal of a single bearing, *x*_*i*_ represents the signal sample at the *i*-th time step, where $\begin{array}{c} x_{i}=(\begin{array}{cc} x_{i}^{1}, & x_{i}^{2},\cdots ,x_{i}^{m} \end{array})T \end{array}$ and *m* denotes the number of data points in a single sample. The signal of a single bearing is represented as $\begin{array}{c} \left(x_{1},x_{2},\cdots ,x_{n}\right) \end{array}$, where *n* denotes the number of samples of the bearing. The model takes consecutive raw time-domain signals as input, with each input sample containing a number of continuous signal samples equal to the time step (timestep). If the inputs are sequential, then the input for the first sample is $\begin{array}{c} X_{1}=(x_{1},x_{2},\cdots ,x_{timestep}) \end{array}$ and the input for the second sample is $\begin{array}{c} X_{2}=(x_{2},x_{3},\cdots ,x_{1+timestep}) \end{array}$. By analogy, the subsequent sample inputs are $\begin{array}{c} X_{i}=(x_{i},x_{i+1},\cdot \cdot \cdot ,x_{i-1+timestep}) \end{array}$ unti $\left(x_{m-timestep},x_{m-timestep+2},\cdots ,x_{m}\right)$is reached [38].

To achieve higher RUL prediction accuracy, this study normalizes the RUL values for each sample point of the bearings [39]. For example, in Condition 1, Bearing No. 6 has a total of 2302 samples and a full-life cycle of 23020 seconds. Therefore, at the 1500th sample point, the corresponding RUL is 8020 seconds, and its normalized value is: RUL1500 = 8020/23020 ≈ 0.3484.

#### 4.2.2 Evaluation metrics.

Equipment degradation is usually caused by very small local defects. After RUL prediction, to more comprehensively evaluate the model’s performance, we use common evaluation metrics such as Mean Absolute Error (MAE), Mean Squared Error (MSE), Root Mean Squared Error (RMSE), and the Score function. The formulas for these four evaluation metrics are as follows:


$$MAE=\frac{1}{n}\sum_{i=1}^{n}\;|y_{i}-{\hat{y}}_{i}|$$ (21)

$$MSE=\frac{1}{n}\sum_{i=1}^{n}\;(y_{i}-{\hat{y}}_{i}{)}^{2}$$ (22)

$$RMSE=\sqrt{\frac{1}{n}\sum_{i=1}^{n}\;(y_{i}-{\hat{y}}_{i}{)}^{2}}$$ (23)

$$Error_{i}=\frac{\left(RUL_{i}^{r}-RUL_{i}^{p}\right)}{RUL_{i}^{r}}\cdot 100\;\%$$ (24)

$$Score=\sum_{i=1}^{m}\;(A_{i})/m$$ (25)

$$Score=\sum_{i=1}^{m}\;(A_{i})/m$$ (26)

Where $y_{i}$ represents the actual data value, $\hat{y}$ represents the predicted data value, n represents the number of samples (i=1,2,⋯n), $RUL_{i}^{r}$ is the actual RUL value of the bearing, $RUL_{i}^{p}$ is the predicted RUL value of the bearing, and $Error_{i}$ is the percentage error of the *i*-th sample.

### 4.3 Experimental setup

To optimize the hyperparameter selection, this study individually adjusted the number of training epochs, learning rate, and batch size, evaluating the resulting prediction performance based on MAE and RMSE metrics. The optimal hyperparameter values were determined accordingly. Table 4 presents the MAE and RMSE results of the BiLSTM-CBAM* model under different hyperparameter settings for the RUL prediction of Bearing 2_4.

**Table 4:** Hyperparameters table. The hyperparameter tuning process of the proposed model.

No.	Training epochs	MAE	RMSE	Learning rate	MAE	RMSE	Batch size	MAE	RMSE
1	50	0.164	0.184	**0.0001**	**0.040**	**0.055**	32	0.051	0.063
2	100	0.628	0.084	0.0002	0.046	0.062	64	0.042	0.056
3	**200**	**0.040**	**0.055**	0.0005	0.053	0.073	**128**	**0.040**	**0.052**
4	300	0.047	0.057	0.001	0.071	0.085	256	0.061	0.083
5	500	0.112	0.133	0.01	0.089	0.108	512	0.070	0.093

As shown in Table 4. By comparing the RUL prediction errors under different parameter settings, we set the number of training epochs to 200, the learning rate to 0.0001, and the batch size to 128. Additionally, the ratio of the training set to the test set is set to 2:1. The mean squared error (MSE) is used as the loss function for all models during training. The experiments were conducted in a software environment consisting of Python 3.8 and TensorFlow 2.6.0, with training performed entirely on a CPU.

### 4.4 Experimental results comparison and analysis

#### 4.4.1 Analysis of BWO-Optimized VMD denoising results.

**Fig 11:**
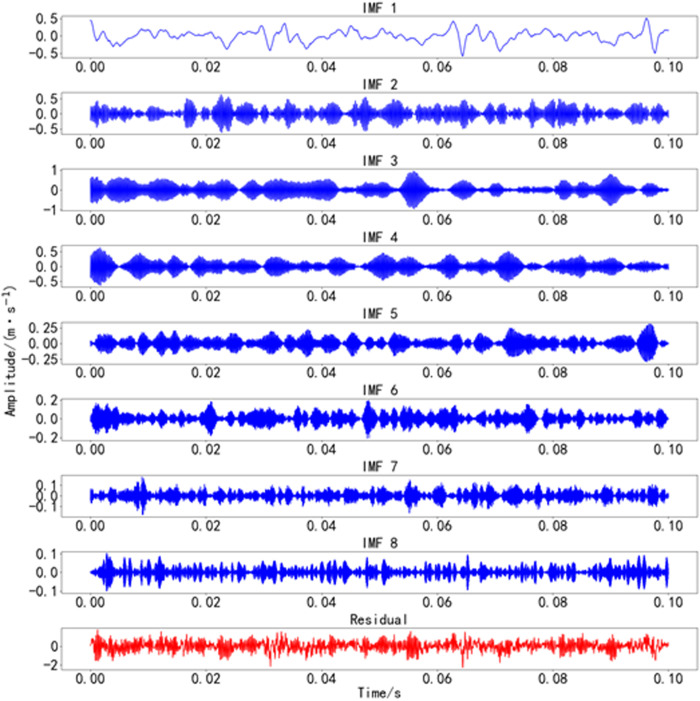
Time-domain decomposition results of Bearing 1_2. Time-domain decomposition results of bearing 1_2 vibration data using BWO-VMD.

**Fig 12:**
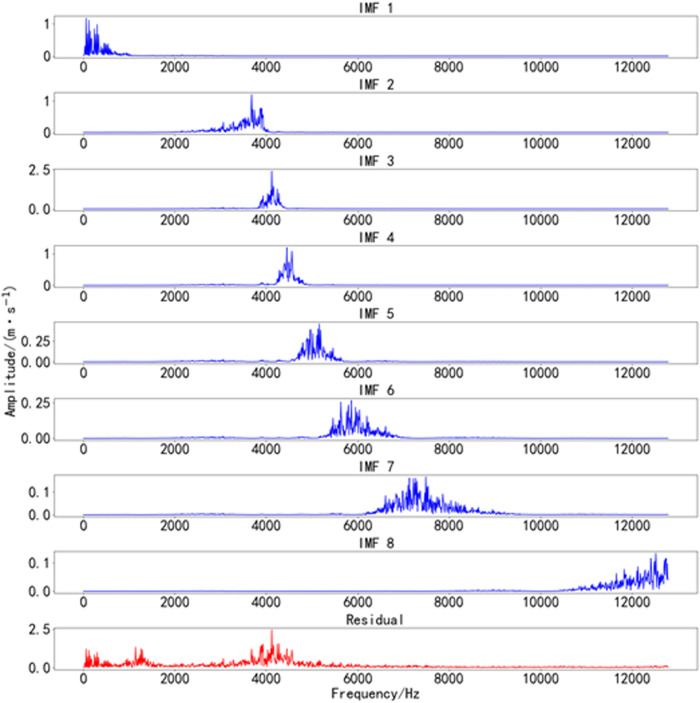
Frequency-domain decomposition results of Bearing 1_2. Frequency-domain decomposition results of bearing 1_2 vibration data using BWO-VMD.

The optimal values found by the BWO optimization algorithm are k=8 and α=577. The time-domain decomposition result of Bearing 1_2 is shown in Fig 11, and the frequency-domain decomposition result of Bearing 1_2 is shown in Fig 12.

As shown in Fig 11 and Fig 12. The vibration signals were decomposed into 8 IMF components using the BWO-optimized VMD algorithm. This method effectively separates the frequency components with minimal under-decomposition or modal aliasing. Subsequently, we calculated the effective kurtosis for each IMF component after decomposition. Based on the effective kurtosis criterion, components with positive effective kurtosis were selected for signal reconstruction.

**Fig 13:**
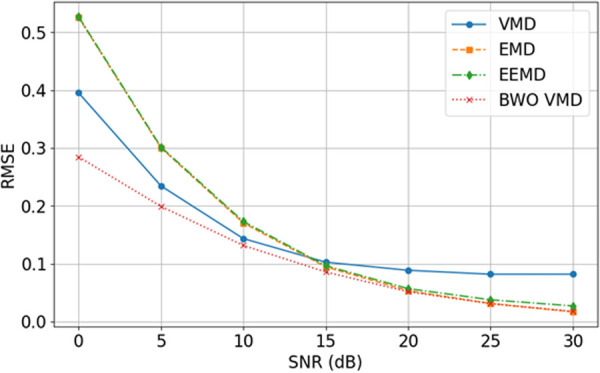
Comparison of Signal Decomposition. RMSE values of decomposition under different signal-to-noise ratios (SNRs) using four methods: VMD, EMD, EEMD, and BWO-VMD.

To validate the superiority of the proposed optimization algorithm’s denoising effect, a comparison is made with VMD, EMD, and EEMD. The RMSE values between the reconstructed signal and the original signal are used as the evaluation metric for the denoising effect of the four algorithms. The RMSE values for bearing 1_2 vibration signal at different signal-to-noise ratios (SNR) are shown in Fig 13.

From Fig 13. It can be seen that as SNR increases, the RMSE gradually decreases. Compared to the VMD, EMD, and EEMD decomposition methods, the BWO-optimized VMD decomposition method exhibits superior noise reduction capability.

#### 4.4.2 Bearing RUL prediction results analysis.

1. comparative experiment

To verify the effectiveness of the method proposed in this study, bearings 2_2 and 2_3 were used as the training set, and bearing 2_4 was used as the test set. The RUL prediction curves of BiLSTM-CBAM* were compared with those of BiGRU-CBAM, as well as the methods presented in References [40] and [41].

**Fig 14:**
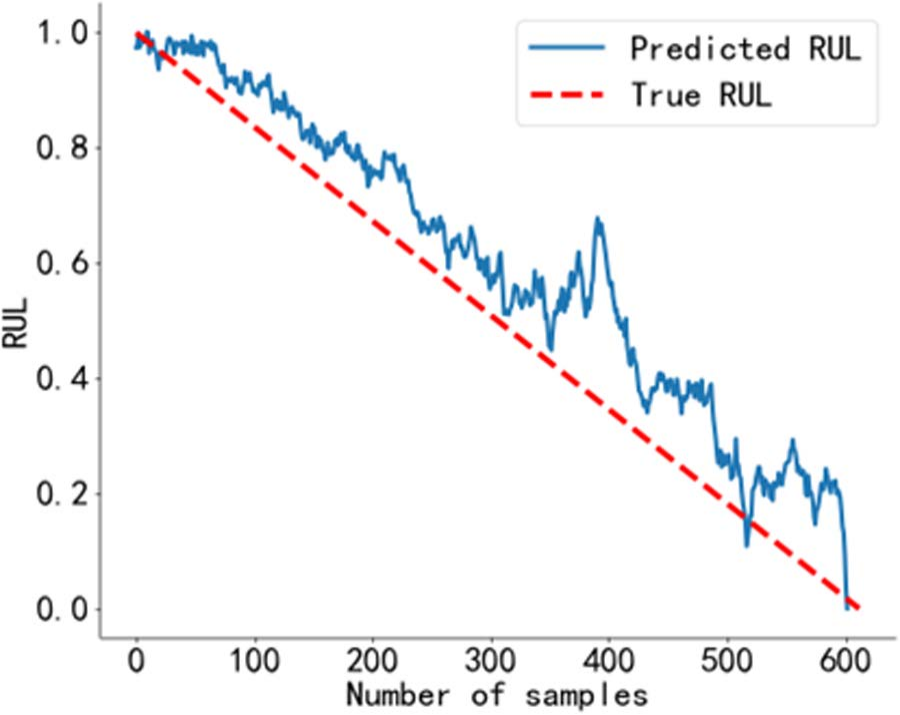
RUL prediction results using the BiGRU-CBAM method. Predicted RUL curve of bearing 2_4 obtained using the BiGRU-CBAM method.

**Fig 15:**
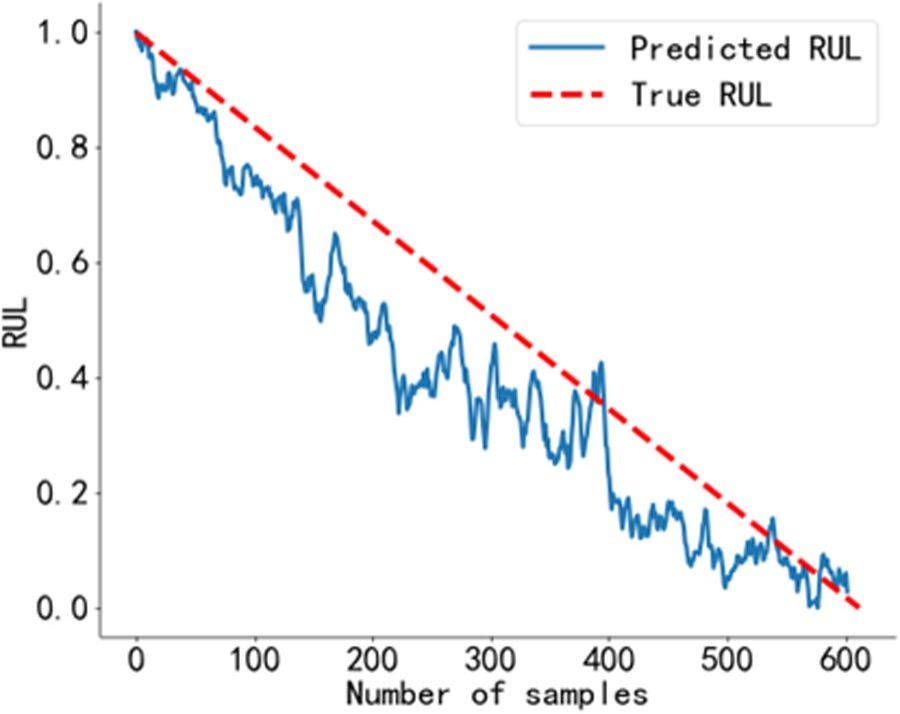
RUL prediction results using the method in Reference [40]. Predicted RUL curve of bearing 2_4 obtained using the method from Reference [40].

**Fig 16:**
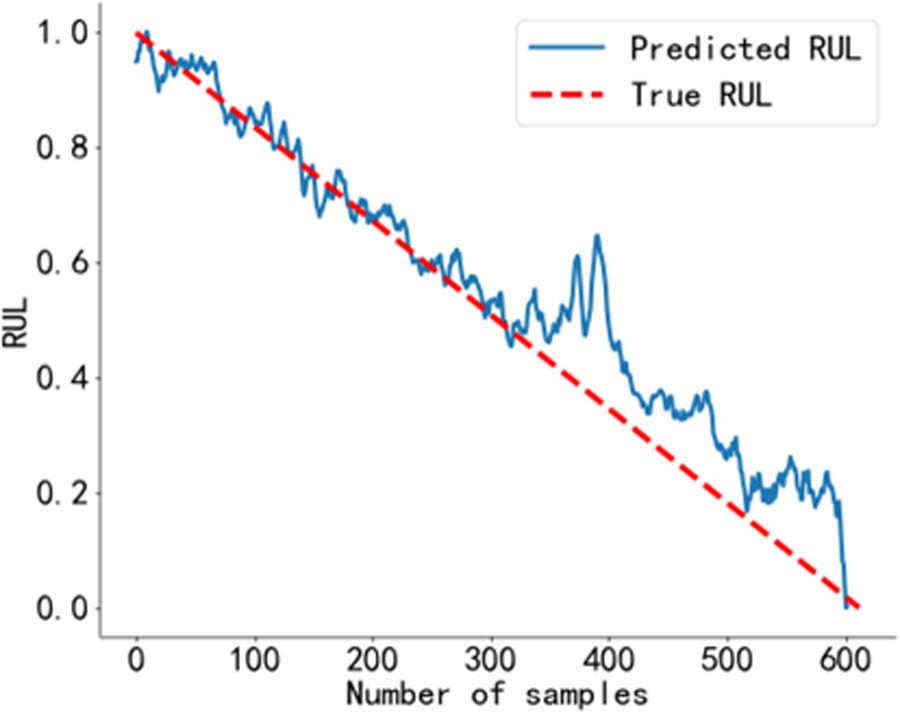
RUL prediction results using the method in Reference [41]. Predicted RUL curve of bearing 2_4 obtained using the method from Reference [41].

**Fig 17:**
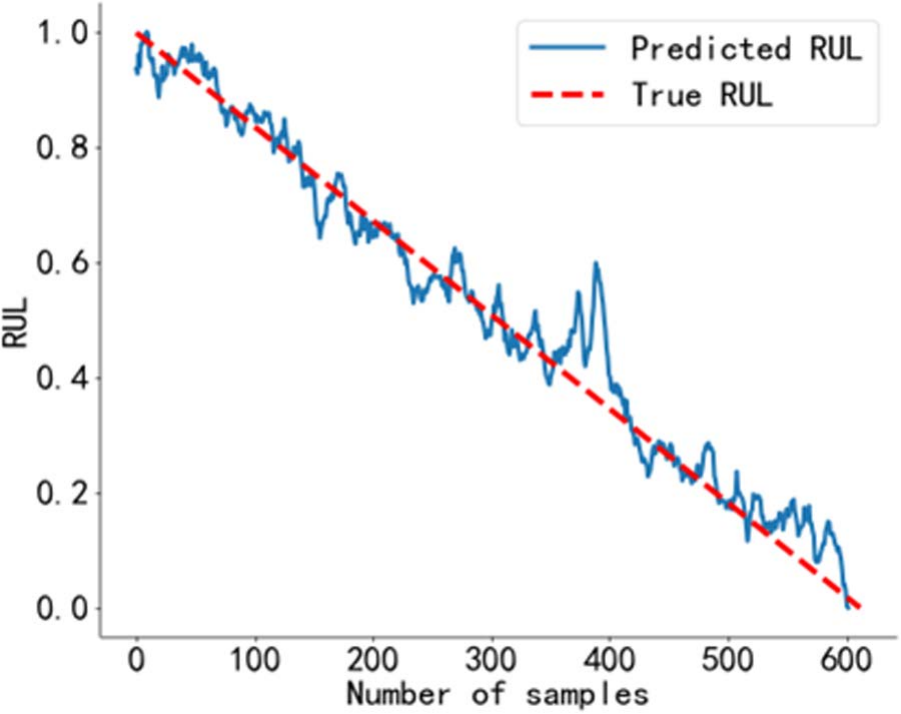
RUL prediction results using the method BWO VMD-BiLSTM-CBAM*. Predicted RUL curve of bearing 2_4 obtained using the method proposed in this study.

The RUL prediction curve using the BiGRU-CBAM method is shown in Fig 14. The RUL prediction curve using the method from Reference [40] is shown in Fig 15, and that using the method from Reference [41] is shown in Fig 16. The RUL prediction curve using the BWO-VMD-BiLSTM-CBAM* method is shown in Fig 17.

As shown in Fig 14 to 17. Compared to the three baseline methods, the RUL prediction values of the BWO VMD-BiLSTM-CBAM* method exhibited the highest degree of alignment with the true values, with no significant fluctuations.

The evaluation of the prediction results for the four methods is shown in Table 5.

**Table 5:** Evaluation of bearing 2_4 prediction results of different models. Comparative experiments with other modeling approaches.

Model/ Evaluation Metrics	MAE	MSE	RMSE	Score
BiGRU-CBAM	0.064	0.006	0.081	0.920
Reference [40]	0.093	0.012	0.112	0.843
Reference [41]	0.053	0.005	0.070	0.937
BWO VMD-BiLSTM-CBAM*	**0.040**	**0.003**	**0.055**	**0.961**

As shown in Table 5. Compared to the other methods—BiGRU-CBAM and the methods from References [40] and [41]—the proposed method achieves a reduction in both MAE and RMSE to varying degrees in the RUL prediction of bearing 2_4. These improvements in both error metrics validate the superiority of the rolling bearing RUL prediction method proposed in this study.

2. ablation study

To verify the effectiveness of the proposed method for RUL prediction of rolling bearings, bearings 2_2 and 2_3 were selected as the training set, while bearings 1_1 and 1_6 were used as the test set under operating condition 1. Under operating condition 2, bearings 2_2 and 2_3 were again used as the training set, and bearings 2_6 and 2_7 were selected as the test set.

**Fig 18:**
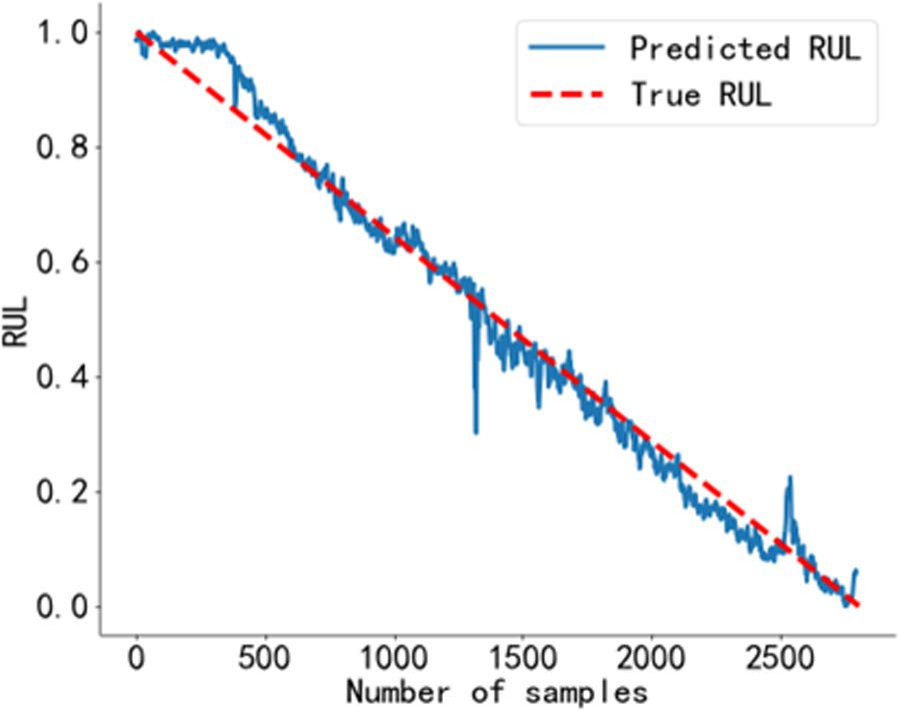
RUL prediction curve of Bearing 1_1. Predicted RUL curve of bearing 1_1 obtained using the method proposed in this study.

**Fig 19:**
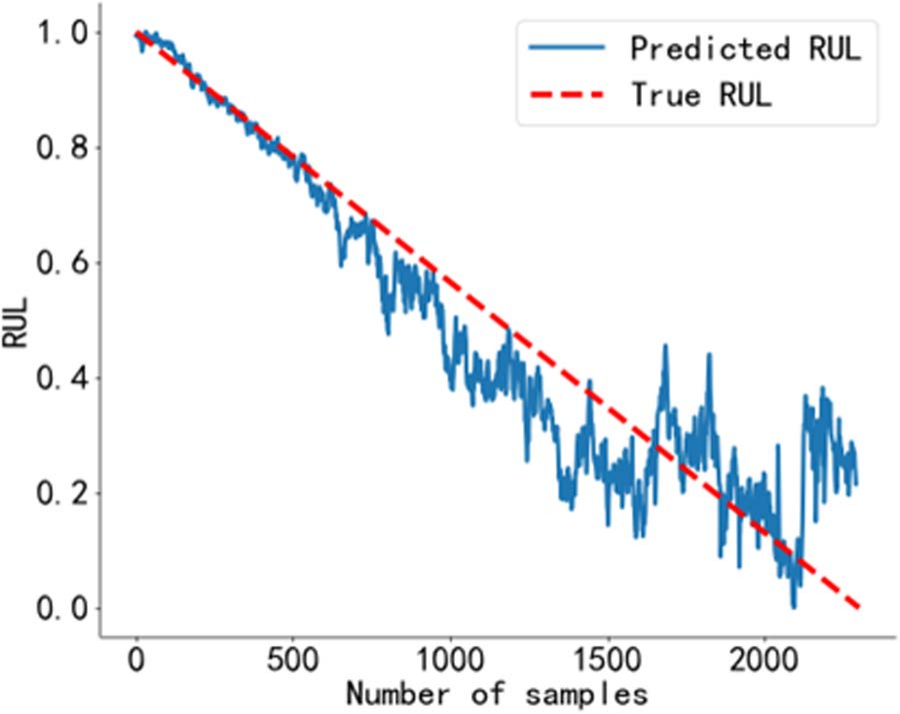
RUL prediction curve of Bearing 1_6. Predicted RUL curve of bearing 1_6 obtained using the method proposed in this study.

**Fig 20:**
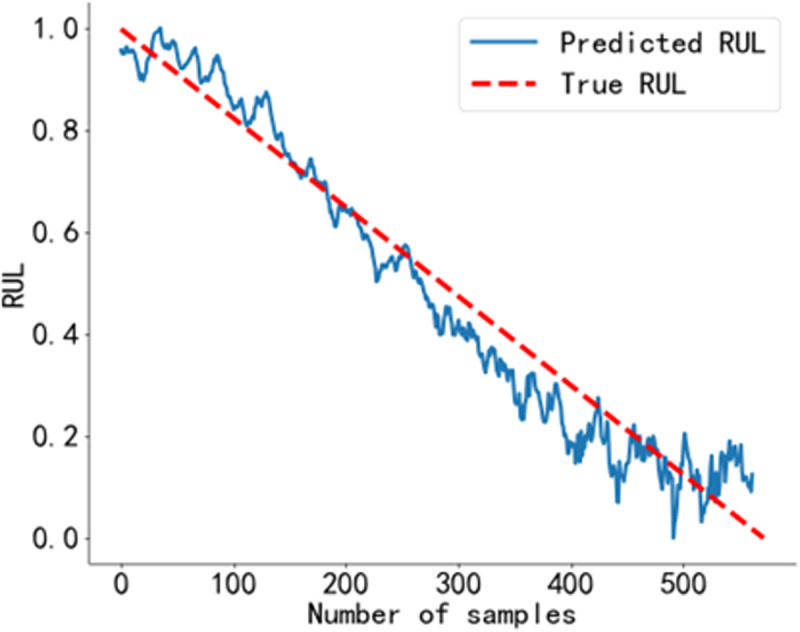
RUL prediction curve of Bearing 2_6. Predicted RUL curve of bearing 2_6 obtained using the method proposed in this study.

**Fig 21:**
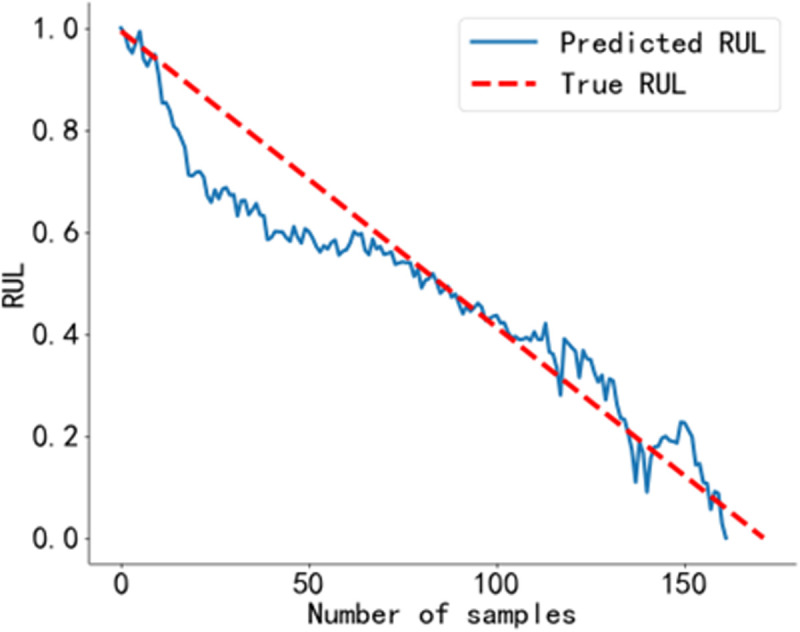
RUL prediction curve of Bearing 2_7. Predicted RUL curve of bearing 2_7 obtained using the method proposed in this study.

The RUL prediction curve of Bearing 1_1 is shown in Fig 18, that of Bearing 1_6 is shown in Fig 19, that of Bearing 2_6 is shown in Fig 20, and that of Bearing 2_7 is shown in Fig 21.

As shown in Fig 18 to 21. The BiLSTM-CBAM* model achieves a high degree of agreement between the predicted and actual RUL values for other rolling bearings, with no significant fluctuations. This suggests that in practical engineering applications, the predicted values can closely approximate the actual values.

To further validate the effectiveness of the proposed method, we conducted three sets of ablation experiments for comparison. The MAE and RMSE error values for the RUL predictions of the aforementioned sets of bearings are shown in Table 6.

**Table 6:** Errors in RUL prediction for different operating conditions. Ablation experiments of the proposed method.

Comparison Models	Evaluation Metrics	Bearing1_1	Bearing1_6	Bearing 2_6	Bearing 2_7
BiLSTM-CBAM	MAE	0.077	0.096	0.101	0.084
	RMSE	0.091	0.122	0.120	0.099
VMD-BiLSTM-CBAM	MAE	0.067	0.085	0.096	0.080
	RMSE	0.078	0.116	0.117	0.097
VMD-BiLSTM-CBAM*	MAE	0.059	0.081	0.071	0.067
	RMSE	0.070	0.112	0.082	0.081
BWO VMD-BiLSTM-CBAM*	**MAE**	**0.038**	**0.070**	**0.049**	**0.059**
	**RMSE**	**0.046**	**0.103**	**0.060**	**0.075**

As shown in Table 6. Compared with the BiLSTM-CBAM model, the VMD-BiLSTM-CBAM model introduces VMD-based decomposition and denoising. The reduction in both MAE and RMSE confirms the effectiveness of decomposition and denoising reconstruction in RUL prediction. The further reduction in MAE and RMSE for VMD-BiLSTM-CBAM* compared to VMD-BiLSTM-CBAM validates the effectiveness of the improved CBAM*. Additionally, the BWO VMD-BiLSTM-CBAM* model incorporates BWO-based parameter optimization, and its lower MAE and RMSE values demonstrate the effectiveness of BWO in optimizing parameters.

Through the above ablation experiments, the proposed BWO VMD-BiLSTM-CBAM* method achieves the lowest MAE and RMSE values, verifying the effectiveness of the constructed approach.

In addition, to validate the effectiveness of degradation feature set construction in bearing RUL prediction, we used the presence or absence of degradation feature set construction as a variable to predict the RUL of the bearings used in this study. We compared the MAE and RMSE prediction errors, and the results are shown in Table 7.

**Table 7:** Comparison of RUL prediction errors before and after degradation feature set construction. Validation experiments on the limitations of constructing the degradation feature set.

Bearing	Evaluation Metric	Without Degradation Feature Set Construction	With Degradation Feature Set Construction
1_1	MAE/RMSE	0.059/0.068	**0.038/0.046**
1_6	MAE/RMSE	0.082/0.115	**0.070/0.103**
2_4	MAE/RMSE	0.082/0.109	**0.040/0.055**
2_6	MAE/RMSE	0.093/0.119	**0.049/0.060**
2_7	MAE/RMSE	0.089/0.106	**0.059/0.075**

As shown in Table 7. After constructing the degradation feature set and removing the time-frequency domain features that poorly reflect the bearing degradation trend, the prediction error of the bearing RUL was reduced, verifying the effectiveness of degradation feature set construction in rolling bearing RUL prediction.

## 5. Conclusion

This study proposes a remaining useful life (RUL) prediction method for rolling bearings based on optimized variational mode decomposition (VMD) and a bi-directional long short-term memory (BiLSTM) network incorporating an improved convolutional attention module. First, the original vibration signals are decomposed using a combination of the Beluga Whale Optimization(BWO) algorithm and the VMD algorithm. Compared to traditional decomposition methods, the proposed decomposition and reconstruction approach demonstrates superior denoising capability.Next, a novel comprehensive evaluation method for time–frequency domain features is defined to assess the extracted time-domain and frequency-domain features. This evaluation is used to construct a degradation feature set by filtering out features that weakly reflect the bearing degradation process. As a result, the computational complexity of the network is reduced, and the prediction accuracy of the bearing RUL is improved. Furthermore, the incorporation of the improved convolutional attention module effectively reduces the RUL prediction error of rolling bearings.

Comparative and ablation experiments were conducted using a public dataset. In the RUL prediction of bearing 2_4, the proposed method achieved the lowest prediction error compared to three other methods, demonstrating its superiority in rolling bearing RUL prediction. In the ablation experiments on bearings 1_1, 1_6, 2_6, and 2_7, the proposed method again yielded the lowest prediction errors, further validating the effectiveness of the approach. An experiment using the presence or absence of degradation feature set construction as a variable showed that constructing the degradation feature set significantly enhances the effectiveness of RUL prediction for rolling bearings.This study is of great significance for the RUL prediction of rolling bearings and also provides valuable reference and guidance for improving the reliability and safety of mechanical equipment.

This paper conducts research on the remaining useful life (RUL) prediction of rolling bearings and achieves promising results. However, all experiments are conducted under fixed operating conditions. In practice, the load and rotational speed of rolling bearings may change during the process from fault occurrence to complete failure. Therefore, future work can focus on RUL prediction of rolling bearings under variable operating conditions.
